# Poly[[tris­(μ_3_-2-oxidopyridinium-3-carboxyl­ato)manganese(II)sodium(I)] monohydrate]

**DOI:** 10.1107/S1600536810002953

**Published:** 2010-01-30

**Authors:** Bing-Yu Zhang, Jing-Jing Nie, Duan-Jun Xu

**Affiliations:** a Department of Chemistry, Zhejiang University, Hangzhou 310027, People’s Republic of China

## Abstract

In the crystal structure of the title compound, {[MnNa(C_6_H_4_NO_3_)_3_]·H_2_O}_*n*_, the Mn^II^ cation is located on a threefold rotation axis and is chelated by three 2-oxidopyridinium-3-carboxyl­ate (opc) anions in an octa­hedal coordination. The Na^I^ cation is located on a threefold rotation axis and is surrounded by six O atoms from three opc anions. The opc anions link the Mn and Na cations, forming a three-dimensional polymeric structure. The uncoordinated water mol­ecule, located on a threefold rotation axis, is equally disordered over two sites. The three-dimensional network is consolidated by N—H⋯O hydrogen bonds.

## Related literature

For related Ni^II^ and Co^II^ complexes, see: Zhang *et al.* (2009*a* ▶,*b* ▶). For comparison C—O bond distances in 2-oxidopyridinium-3-carboxyl­ate and 2-hydroxy­pyridine­carboxyl­ate complexes, see: Yao *et al.* (2004 ▶); Yan & Hu (2007*a* ▶,*b* ▶); Wen & Liu (2007 ▶); Quintal *et al.* (2002 ▶). For comparison C—O bond distances in 2-hydroxy­benzoic acid and 2-hydroxy­benzoate complexes, see: Munshi & Guru Row (2006 ▶); Su & Xu (2005 ▶); Li *et al.* (2005 ▶). 
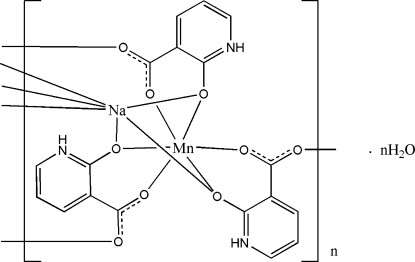


## Experimental

### 

#### Crystal data

[MnNa(C_6_H_4_NO_3_)_3_]·H_2_O
*M* *_r_* = 510.25
Trigonal, 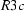

*a* = 10.1478 (18) Å
*c* = 37.420 (13) Å
*V* = 3337.1 (15) Å^3^
*Z* = 6
Mo *K*α radiation
μ = 0.67 mm^−1^
*T* = 294 K
0.33 × 0.28 × 0.26 mm

#### Data collection

Rigaku R-AXIS RAPID IP diffractometer
Absorption correction: multi-scan (*ABSCOR*; Higashi, 1995 ▶) *T*_min_ = 0.822, *T*_max_ = 0.840
6825 measured reflections
1315 independent reflections
1236 reflections with *I* > 2σ(*I*)
*R*_int_ = 0.027

#### Refinement

*R*[*F* ^2^ > 2σ(*F* ^2^)] = 0.033
*wR*(*F* ^2^) = 0.099
*S* = 1.16
1315 reflections
103 parameters
1 restraint
H-atom parameters constrained
Δρ_max_ = 0.35 e Å^−3^
Δρ_min_ = −0.49 e Å^−3^
Absolute structure: Flack (1983 ▶), 649 Friedel pairs
Flack parameter: −0.01 (3)

### 

Data collection: *PROCESS-AUTO* (Rigaku, 1998 ▶); cell refinement: *PROCESS-AUTO*; data reduction: *CrystalStructure* (Rigaku/MSC, 2002 ▶); program(s) used to solve structure: *SIR92* (Altomare *et al.*, 1993 ▶); program(s) used to refine structure: *SHELXL97* (Sheldrick, 2008 ▶); molecular graphics: *ORTEP-3* (Farrugia, 1997 ▶); software used to prepare material for publication: *WinGX* (Farrugia, 1999 ▶).

## Supplementary Material

Crystal structure: contains datablocks I, global. DOI: 10.1107/S1600536810002953/ng2724sup1.cif

Structure factors: contains datablocks I. DOI: 10.1107/S1600536810002953/ng2724Isup2.hkl

Additional supplementary materials:  crystallographic information; 3D view; checkCIF report

## Figures and Tables

**Table 1:** Selected bond lengths (Å)

Mn—O2	2.123 (3)
Mn—O3	2.168 (2)
Na1—O1^i^	2.331 (2)
Na1—O3	2.459 (3)

Symmetry code: (i) 
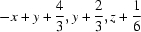
.

**Table 2:** Hydrogen-bond geometry (Å, °)

*D*—H⋯*A*	*D*—H	H⋯*A*	*D*⋯*A*	*D*—H⋯*A*
N1—H1*N*⋯O1^ii^	0.90	2.12	2.983 (4)	161
N1—H1*N*⋯O2^ii^	0.90	2.37	3.113 (4)	140

Symmetry code: (ii) 
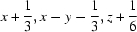
.

**Fig. 1.**
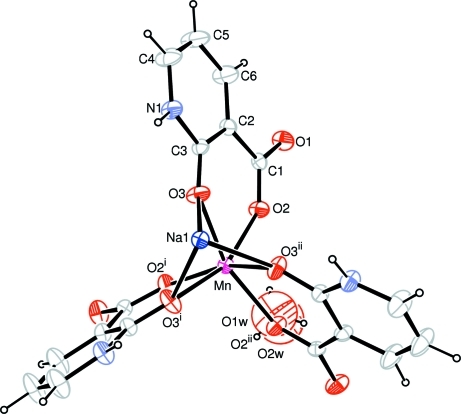
The coordination environment around a Mn cation and a Na cation with 30% probability displacement ellipsoids (arbitrary spheres for H atoms) [symmetry codes: (i) 1-y, x-y, z; (ii) 1-x+y, 1-x, z].

## supplementary crystallographic information

### Comment

As a part of ongoing investigation on π-π stacking (Li *et al.*, 2005), the title complex has been prepared in the laboratory and its crystal structure is reported here.

In the crystal structure the Mn^II^ cation is located in a three-fold ratation axis and is chelated by three 2-oxidopyridinium-3-carboxylate (opc) anions in a distorted anti-triprism geometry (Fig. 1). The Na^I^ cation is located on the same three-fold rotation axis and is surrounded by six O atoms from three opc anions (Table 1). The opc anions link the Mn and Na cations to form the three dimensional polymeric structure.

The shorter C—O bond distance of 1.251 (4) Å is observed between the deprotonated hydroxy group and pyridinium ring. This is similar to those found in the related complexes of oxidopyridinium-carboxylate (Yao *et al.*, 2004; Yan & Hu, 2007a,b; Wen & Liu, 2007; Zhang *et al.* 2009a,b), it is also consistent with that found in hydroxy-pyridinecarboxylate complex (Quintal *et al.* 2002). This finding suggests the electron delocalization between pyridine ring and hydroxy group. But this shorter C—O bond is much different from the C—O bond distance of ca. 1.35 Å between benzene ring and hydroxy-O atom found in hydroxy-benzencarboxylic acid (Munshi & Guru Row, 2006) and in hydroxy-benzenecarboxylate complexes of metals (Su & Xu, 2005; Li *et al.*, 2005).

The lattice water molecule located on the three-fold rotation axis is disordered over two sites with o.5 occupancies for each component. The N—H···O hydrogen bondings are present in the polymeric structure. No π-π stacking is observed in the crystal structure.

### Experimental

2-Hydroxy-pyridine-3-carboxylic acid (0.13 g, 1 mmol), NaOH (0.04 g, 1 mmol), imidazole (0.14 g, 2 mmol) and Mn(NO_3_)_2_ (0.18 g, 1 mmol) and water (8 ml) and ethanol (2 ml) were sealed in a 25 ml stainless steel reactor with a Teflon liner. The reaction system was heated at 433 K for 9 h. After the mixture was cooled to room temperature the single crystals of the title complex were obtained.

### Refinement

The lattice water molecule is disordered over two sites with 0.5 occupancy for each component, the water H atom was placed in a chemical sensitive position and refined in a riding mode with U_iso_(H) = 1.2U_eq_(O1W). The H atom bonded to the pyridine N was located in a difference Fourier map and refined as riding in as-found relative position with U_iso_(H) = 1.2U_eq_(N). Other H atoms were placed in calculated positions with C—H = 0.93 and refined in riding mode with U_iso_(H) = 1.2U_eq_(C).

### Crystal data

**Table:**

[MnNa(C_6_H_4_NO_3_)_3_]·H_2_O	*D*_x_ = 1.523 Mg m^−^^3^
*M**_r_* = 510.25	Mo *K*α radiation, λ = 0.71073 Å
Trigonal, *R*3*c*	Cell parameters from 1286 reflections
Hall symbol: R 3 -2"c	θ = 2.6–25.0°
*a* = 10.1478 (18) Å	µ = 0.67 mm^−^^1^
*c* = 37.420 (13) Å	*T* = 294 K
*V* = 3337.1 (15) Å^3^	Prism, brown
*Z* = 6	0.33 × 0.28 × 0.26 mm
*F*(000) = 1554	

### Data collection

**Table:**

Rigaku R-AXIS RAPID IP diffractometer	1315 independent reflections
Radiation source: fine-focus sealed tube	1236 reflections with *I* > 2σ(*I*)
graphite	*R*_int_ = 0.027
Detector resolution: 10.00 pixels mm^-1^	θ_max_ = 25.2°, θ_min_ = 2.6°
ω scan	*h* = −11→11
Absorption correction: multi-scan (*ABSCOR*; Higashi, 1995)	*k* = −11→11
*T*_min_ = 0.822, *T*_max_ = 0.840	*l* = −44→44
6825 measured reflections	

### Refinement

**Table:**

Refinement on *F*^2^	Secondary atom site location: difference Fourier map
Least-squares matrix: full	Hydrogen site location: inferred from neighbouring sites
*R*[*F*^2^ > 2σ(*F*^2^)] = 0.033	H-atom parameters constrained
*wR*(*F*^2^) = 0.099	*w* = 1/[σ^2^(*F*_o_^2^) + (0.0683*P*)^2^ + 0.0175*P*] where *P* = (*F*_o_^2^ + 2*F*_c_^2^)/3
*S* = 1.16	(Δ/σ)_max_ = 0.001
1315 reflections	Δρ_max_ = 0.35 e Å^−^^3^
103 parameters	Δρ_min_ = −0.49 e Å^−^^3^
1 restraint	Absolute structure: Flack (1983), 649 Friedel pairs
Primary atom site location: structure-invariant direct methods	Flack parameter: −0.01 (3)

### Special details

**Table:**

Geometry. All esds (except the esd in the dihedral angle between two l.s. planes) are estimated using the full covariance matrix. The cell esds are taken into account individually in the estimation of esds in distances, angles and torsion angles; correlations between esds in cell parameters are only used when they are defined by crystal symmetry. An approximate (isotropic) treatment of cell esds is used for estimating esds involving l.s. planes.
Refinement. Refinement of *F*^2^ against ALL reflections. The weighted *R*-factor wR and goodness of fit *S* are based on *F*^2^, conventional *R*-factors *R* are based on *F*, with *F* set to zero for negative *F*^2^. The threshold expression of *F*^2^ > σ(*F*^2^) is used only for calculating *R*-factors(gt) etc. and is not relevant to the choice of reflections for refinement. *R*-factors based on *F*^2^ are statistically about twice as large as those based on *F*, and *R*- factors based on ALL data will be even larger.

### Fractional atomic coordinates and isotropic or equivalent isotropic displacement parameters (Å^2^)

**Table:**

	*x*	*y*	*z*	*U*_iso_*/*U*_eq_	Occ. (<1)
Mn	0.6667	0.3333	0.87519 (2)	0.0258 (2)	
Na1	0.6667	0.3333	0.96375 (5)	0.0283 (5)	
N1	0.7336 (3)	0.0138 (3)	0.93953 (7)	0.0401 (6)	
H1N	0.7655	0.0750	0.9589	0.048*	
O1	0.4925 (3)	−0.1044 (3)	0.82611 (6)	0.0464 (6)	
O2	0.5735 (3)	0.1281 (3)	0.84538 (6)	0.0377 (6)	
O3	0.6999 (3)	0.1941 (3)	0.91404 (6)	0.0425 (6)	
C1	0.5566 (3)	−0.0028 (3)	0.84910 (7)	0.0292 (6)	
C2	0.6173 (3)	−0.0412 (3)	0.88206 (8)	0.0321 (6)	
C3	0.6828 (3)	0.0635 (3)	0.91133 (8)	0.0293 (6)	
C4	0.7283 (6)	−0.1207 (4)	0.94099 (12)	0.0596 (12)	
H4	0.7664	−0.1454	0.9609	0.072*	
C5	0.6677 (6)	−0.2210 (4)	0.91359 (12)	0.0689 (13)	
H5	0.6651	−0.3139	0.9141	0.083*	
C6	0.6092 (6)	−0.1799 (4)	0.88440 (12)	0.0581 (12)	
H6	0.5631	−0.2494	0.8659	0.070*	
O1W	0.6667	0.3333	0.6735 (14)	0.26 (3)	0.50
H1W	0.5895	0.3288	0.6601	0.310*	0.6667
O2W	0.6667	0.3333	0.6459 (14)	0.30 (3)	0.50

### Atomic displacement parameters (Å^2^)

**Table:**

	*U*^11^	*U*^22^	*U*^33^	*U*^12^	*U*^13^	*U*^23^
Mn	0.0289 (3)	0.0289 (3)	0.0195 (4)	0.01446 (13)	0.000	0.000
Na1	0.0312 (7)	0.0312 (7)	0.0225 (11)	0.0156 (4)	0.000	0.000
N1	0.0555 (16)	0.0401 (14)	0.0283 (13)	0.0266 (13)	−0.0171 (12)	−0.0075 (10)
O1	0.0624 (15)	0.0393 (12)	0.0297 (12)	0.0195 (12)	−0.0221 (12)	−0.0113 (10)
O2	0.0536 (14)	0.0337 (13)	0.0250 (11)	0.0213 (9)	−0.0148 (10)	−0.0030 (10)
O3	0.0703 (17)	0.0398 (13)	0.0283 (12)	0.0358 (13)	−0.0199 (12)	−0.0107 (11)
C1	0.0288 (14)	0.0302 (16)	0.0235 (13)	0.0110 (12)	−0.0031 (12)	−0.0034 (12)
C2	0.0360 (14)	0.0296 (14)	0.0269 (15)	0.0135 (13)	−0.0089 (12)	−0.0046 (12)
C3	0.0348 (15)	0.0314 (15)	0.0238 (13)	0.0180 (13)	−0.0069 (11)	−0.0005 (11)
C4	0.094 (3)	0.050 (2)	0.044 (2)	0.043 (2)	−0.031 (2)	−0.0023 (17)
C5	0.113 (4)	0.042 (2)	0.063 (2)	0.047 (3)	−0.041 (2)	−0.011 (2)
C6	0.091 (3)	0.0422 (19)	0.048 (2)	0.038 (2)	−0.034 (2)	−0.0189 (17)
O1W	0.31 (4)	0.31 (4)	0.16 (4)	0.15 (2)	0.000	0.000
O2W	0.34 (5)	0.34 (5)	0.23 (6)	0.17 (2)	0.000	0.000

### Geometric parameters (Å, °)

**Table:**

Mn—O2	2.123 (3)	N1—H1N	0.9033
Mn—O2^i^	2.123 (3)	O1—C1	1.247 (4)
Mn—O2^ii^	2.123 (3)	O2—C1	1.260 (4)
Mn—O3	2.168 (2)	O3—C3	1.251 (4)
Mn—O3^i^	2.168 (2)	C1—C2	1.514 (4)
Mn—O3^ii^	2.168 (2)	C2—C6	1.370 (4)
Mn—Na1	3.314 (2)	C2—C3	1.437 (4)
Na1—O1^iii^	2.331 (2)	C4—C5	1.356 (6)
Na1—O1^iv^	2.331 (3)	C4—H4	0.9300
Na1—O1^v^	2.331 (2)	C5—C6	1.403 (6)
Na1—O3	2.459 (3)	C5—H5	0.9300
Na1—O3^i^	2.459 (3)	C6—H6	0.9300
Na1—O3^ii^	2.459 (3)	O1W—H1W	0.9106
N1—C4	1.339 (5)	O2W—H1W	0.9294
N1—C3	1.376 (4)		
			
O2—Mn—O2^i^	94.91 (9)	O3—Na1—O3^ii^	69.00 (10)
O2—Mn—O2^ii^	94.91 (9)	O3^i^—Na1—O3^ii^	69.00 (10)
O2^i^—Mn—O2^ii^	94.91 (9)	O1^iii^—Na1—Mn	117.77 (8)
O2—Mn—O3	81.45 (8)	O1^iv^—Na1—Mn	117.77 (8)
O2^i^—Mn—O3	105.70 (10)	O1^v^—Na1—Mn	117.77 (8)
O2^ii^—Mn—O3	159.28 (9)	O3—Na1—Mn	40.85 (6)
O2—Mn—O3^i^	159.28 (9)	O3^i^—Na1—Mn	40.85 (6)
O2^i^—Mn—O3^i^	81.45 (8)	O3^ii^—Na1—Mn	40.85 (6)
O2^ii^—Mn—O3^i^	105.70 (10)	C4—N1—C3	125.0 (3)
O3—Mn—O3^i^	79.96 (10)	C4—N1—H1N	119.0
O2—Mn—O3^ii^	105.70 (11)	C3—N1—H1N	115.8
O2^i^—Mn—O3^ii^	159.28 (9)	C1—O1—Na1^vi^	163.8 (2)
O2^ii^—Mn—O3^ii^	81.45 (8)	C1—O2—Mn	137.3 (2)
O3—Mn—O3^ii^	79.96 (10)	C3—O3—Mn	130.64 (19)
O3^i^—Mn—O3^ii^	79.96 (10)	C3—O3—Na1	133.1 (2)
O2—Mn—Na1	121.71 (7)	Mn—O3—Na1	91.26 (9)
O2^i^—Mn—Na1	121.71 (7)	O1—C1—O2	122.4 (3)
O2^ii^—Mn—Na1	121.71 (7)	O1—C1—C2	117.4 (3)
O3—Mn—Na1	47.89 (6)	O2—C1—C2	120.3 (3)
O3^i^—Mn—Na1	47.89 (7)	C6—C2—C3	118.6 (3)
O3^ii^—Mn—Na1	47.89 (6)	C6—C2—C1	119.5 (3)
O1^iii^—Na1—O1^iv^	100.04 (10)	C3—C2—C1	121.8 (2)
O1^iii^—Na1—O1^v^	100.04 (10)	O3—C3—N1	116.6 (3)
O1^iv^—Na1—O1^v^	100.04 (10)	O3—C3—C2	127.8 (3)
O1^iii^—Na1—O3	149.83 (11)	N1—C3—C2	115.6 (2)
O1^iv^—Na1—O3	109.27 (10)	N1—C4—C5	120.4 (4)
O1^v^—Na1—O3	82.12 (8)	N1—C4—H4	119.8
O1^iii^—Na1—O3^i^	82.12 (8)	C5—C4—H4	119.8
O1^iv^—Na1—O3^i^	149.83 (11)	C4—C5—C6	117.8 (3)
O1^v^—Na1—O3^i^	109.27 (10)	C4—C5—H5	121.1
O3—Na1—O3^i^	69.00 (10)	C6—C5—H5	121.1
O1^iii^—Na1—O3^ii^	109.27 (10)	C2—C6—C5	122.5 (4)
O1^iv^—Na1—O3^ii^	82.12 (8)	C2—C6—H6	118.7
O1^v^—Na1—O3^ii^	149.83 (11)	C5—C6—H6	118.7

Symmetry codes: (i) −*y*+1, *x*−*y*, *z*; (ii) −*x*+*y*+1, −*x*+1, *z*; (iii) −*x*+*y*+4/3, *y*+2/3, *z*+1/6; (iv) −*y*+1/3, −*x*+2/3, *z*+1/6; (v) *x*+1/3, *x*−*y*−1/3, *z*+1/6; (vi) −*x*+*y*+2/3, *y*−2/3, *z*−1/6.

### Hydrogen-bond geometry (Å, °)

**Table:**

*D*—H···*A*	*D*—H	H···*A*	*D*···*A*	*D*—H···*A*
N1—H1N···O1^v^	0.90	2.12	2.983 (4)	161
N1—H1N···O2^v^	0.90	2.37	3.113 (4)	140

Symmetry codes: (v) *x*+1/3, *x*−*y*−1/3, *z*+1/6.
